# Navigating the Landscape of B Cell Mediated Immunity and Antibody Monitoring in SARS-CoV-2 Vaccine Efficacy: Tools, Strategies and Clinical Trial Insights

**DOI:** 10.3390/vaccines12101089

**Published:** 2024-09-24

**Authors:** Sophie O’Reilly, Joanne Byrne, Eoin R. Feeney, Patrick W. G. Mallon, Virginie Gautier

**Affiliations:** 1 Centre for Experimental Pathogen Host Research (CEPHR), University College Dublin, Belfield, Dublin 4, Ireland; 2 School of Medicine, University College Dublin, Belfield, Dublin 4, Ireland; 3 Conway Institute of Biomolecular and Biomedical Research, University College Dublin, Belfield, Dublin 4, Ireland; 4 Department of Infectious Diseases, St Vincent’s University Hospital, Elm Park, Dublin 4, Ireland

**Keywords:** SARS-CoV-2, COVID-19, correlates of protections, biomarkers, humoral immunity, immunoglobulins, neutralising antibodies, Fc effector functions, memory B cells, antibody secreting cells (ASC), clinical vaccine trials

## Abstract

Correlates of Protection (CoP) are biomarkers above a defined threshold that can replace clinical outcomes as primary endpoints, predicting vaccine effectiveness to support the approval of new vaccines or follow up studies. In the context of COVID-19 vaccination, CoPs can help address challenges such as demonstrating vaccine effectiveness in special populations, against emerging SARS-CoV-2 variants or determining the durability of vaccine-elicited immunity. While anti-spike IgG titres and viral neutralising capacity have been characterised as CoPs for COVID-19 vaccination, the contribution of other components of the humoral immune response to immediate and long-term protective immunity is less well characterised. This review examines the evidence supporting the use of CoPs in COVID-19 clinical vaccine trials, and how they can be used to define a protective threshold of immunity. It also highlights alternative humoral immune biomarkers, including Fc effector function, mucosal immunity, and the generation of long-lived plasma and memory B cells and discuss how these can be applied to clinical studies and the tools available to study them.

## 1. Introduction

Systemic and mucosal humoral immunity is critical in limiting infection and transmission of SARS-CoV-2, the causative agent of COVID-19. Unprecedented research and funding efforts globally, together with academic and industry coordination have facilitated multiple COVID-19 vaccines to be trialled, authorised and rolled out in a global vaccination campaign. These campaigns have been hugely successful, estimated to have saved 14.4 million lives globally in the first year [1] and 1.4 million lives to date in Europe alone [2]. The primary endpoints for early COVID-19 placebo-controlled vaccine trials were safety and vaccine efficacy outcomes including preventing severe disease [3], hospitalisation, and death after completion of a full vaccine course, as well as interrupting transmission of infection.

Follow up vaccination studies to confirm clinical benefit adapted their strategies, switching from clinical outcomes as primary endpoints to using humoral immune biomarkers as Correlates of Protection (CoP) to examine the effectiveness of the vaccine in ‘real-world’ settings and within representative cohorts. CoPs are measurable immune responses above a defined threshold statistically shown to be associated with protective immunity. For COVID-19, anti-SARS-CoV-2 Spike Immunoglobulin (Ig) titres and the capacity of serum Ig to neutralise SARS-CoV-2 (neutralisation titre) have both been shown to be CoPs, and have been incorporated into clinical trials to demonstrate vaccine efficacy as primary endpoints [4,5,6,7,8,9].

In 2021, The International Coalition of Medicines Regulatory Authorities (ICMRA) agreed that well-justified and appropriately designed immunobridging studies are an acceptable approach for authorising new COVID-19 vaccines if clinical endpoint efficacy studies are no longer feasible [10]. In this instance, clinical immunobridging infers effectiveness of candidate vaccines based on comparable immune CoPs to approved vaccines with known clinical efficacy.

In some early placebo-controlled trials, the use of CoPs was also applied to populations who experienced few clinically significant endpoints. The ‘Teen COVE’ trial, a placebo-controlled evaluation of the safety, immunogenicity and efficacy of mRNA-1273 vaccine in adolescents, selected non-inferiority of neutralisation titres in adolescents as compared to young adults in the ‘COVE’ study as their primary efficacy endpoint. Whilst they also measured vaccine efficacy by comparing disease incidence between the trial arms, this was difficult to assess due to low incidence of COVID-19 infection in the study population [6].

Here we review the variety and diversity of biomarkers available to monitor humoral immunity, focusing on immunoglobulins, their functions, and their relevance and significance to evaluate efficacy of COVID-19 vaccines. We first focus on the two leading CoPs of vaccine-mediated humoral immunity, anti-spike IgG titre and virus neutralisation capacity. We look at the evidence supporting their use as primary endpoints for clinical trials, for establishing a protective threshold of immunity, and examine the tools used to measure them. We will then highlight some alternative humoral immune biomarkers including Fc effector functions, IgA and IgM and discuss their importance to better comprehend and fully characterise COVID-19 vaccine-induced humoral immunity. Finally we examine B cells, the pivotal role of B cell subsets in immediate versus long-term protection and the tools available to assess them.

## 2. Immunoglobulin Response to COVID-19 Vaccination

Immunoglobulins (Ig) are soluble effector molecules released by Antibody Secreting Cells (ASCs), which are terminally differentiated B cells. Ig structure comprises two antibody binding fragments (Fab) opposite to one crystalline fragment (Fc). The former binds to and defines antigen specificity while the latter defines the isotype (IgA, IgD, IgE, IgG or IgM). Also relevant, Fc domain links humoral and cellular innate immune compartments through interaction with the complement system or Fc receptors (FcRs) on innate immune cells, through which they mediate effector functions. These include antibody-dependent cellular cytotoxicity (ADCC), antibody-dependent cellular phagocytosis (ADCP) and antibody-dependent complement deposition (ADCD).

**Figure 1:**
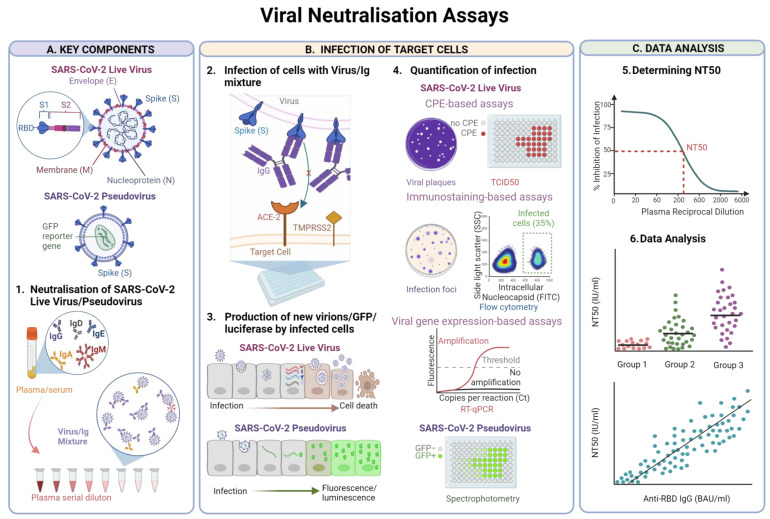
Viral Neutralisation Assays. (**A**) SARS-CoV-2 virion with the four structural proteins: spike, nucleoprotein, envelope and membrane and details of the spike S1 and S2 subunits and the Receptor Binding Domain (RBD). SARS-CoV-2 pseudovirus expressing spike proteins and harbouring a GFP reporter gene. (**A1**) Plasma or serum, containing a variety of Ig is serially diluted and mixed with live virus or pseudovirus facilitating the interactions between Ig and spike. Panel (**B**). (**B2**) Virus/Ig mixture is added to a monolayer of cells. Spike mediates viral entry through interaction between RBD and the ACE2 receptor on the host cell. Spike targeted by neutralising antibodies fail to interact with ACE2 and block viral entry. (**B3**) Within hours, viral replication takes place, leading to formation and release of new virions, ultimately resulting in cell death. Alternatively cells infected with SARS-CoV-2 pseudovirus produce GFP or luciferase encoded by a reporter gene. (**B4**) For live-virus assays, infection can be quantified through observed cytopathic effects (CPE) such as visual counting of plaques or scoring CPE in each well using a light microscope (TCID50). Immunostaining facilitates the detection of viral proteins. Micro-foci can be quantified using spot-readers, or infected cells can be quantified using flow cytometry. RT-qPCR quantifies cell-associated viral RNA or viral load. Spectrophotometry quantifies fluorescence or luminescence in cells infected with SARS-CoV-2 pseudovirus. Panel (**C**). (**C5**) The reciprocal of the plasma dilution that reduces infection rate by 50% (NT50) is determined by normalising the infection rate measured for each dilution to positive (no plasma) and negative (no virus) control wells. (**C6**) The NT50 values can then be compared between cohorts e.g., with different vaccine strategies or against SARS-CoV-2 variants to monitor immune escape. NT50 values can also be correlated with other humoral immune biomarkers such as specific IgG titres. Created with BioRender.com (accessed on 19 September 2024).

During SARS-CoV-2 infection, Ig produced against the four viral structural proteins, spike (S), nucleoprotein (N), envelope (E) and matrix (M) (Figure 1) can be detected in the serum. Most clinical studies monitor S and N antibody levels. The S glycoprotein is a transmembrane protein expressed on the surface of the virion, easily accessible to Ig. Critically, S mediates viral entry into cells through an interaction of its Receptor Binding Domain (RBD) with the angiotensin-converting enzyme-2 (ACE2) receptor on target cells. Ig targeting S and RBD in particular can directly interfere with this crucial step of the viral replication cycle and target virions for elimination (as described in Box 1). For these reasons, S was selected as the antigen of choice for COVID-19 vaccine development.

Box 1 **SARS-CoV-2 Spike Epitopes.**

SARS-CoV-2 virions have an outer surface spike (S) protein which is a homotrimer of glycoproteins comprised of S1 and S2 subunits, connected by a protease cleavage site. S1 contains a receptor binding site (RBD), and an N-terminal domain (NTD). In a pre-fusion state, the three RBDs of S1 are at the apex of the S trimer. S1 can take two distinct conformations, ‘up’ where the RBD is accessible and ‘down’ where RBD is inaccessible. The S2 subunit forms a central helical bundle and is responsible for membrane fusion. Following engagement of RBD with ACE2, the S1 subunit disassociates and the S2 subunit undergoes a conformational change resulting in the exposure of the fusion peptide which is inserted into the host cell membrane, facilitating fusion of viral and cell membranes and release of the viral genomic RNA into the cytosol of the host cell [11].

The SARS-CoV-2 spike protein is heavily glycosylated, with glycans covering roughly 40% of the surface area [12]. Glycans shield the protein from immune recognition and affect protein folding. Mutations in glycosylation sites, particularly across S1 and RBD, have been shown to reduce incorporation of spike into pseudovirus virions and reduce infectivity [13].

Recombinant S protein is prone to shed its S1 subunit, taking its post-fusion conformation [14]. As S1 epitopes including the RBD are highly-immunogenic strategies have been developed to stabilise the S protein in its pre-fusion conformation, primarily the inclusion of two proline substitutions in S2 [15] which have been included in vaccine formulas including BNT162b2 [16], Novavax [17], mRNA-1273 [18], and Ad26 [19]. An alternative approach involves six proline substitutions and has been shown to increase yield, protein stability and immunogenicity [20,21].

The RBD is the primary target of neutralising Abs [22]. Ig targeting the receptor binding motif (RBM) within the RBD can prevent infection by blocking the S protein binding to ACE-2. Ig targeting this site are less likely to be cross-reactive to subsequent SARS-CoV-2 VOCs or other coronaviruses as the site is highly mutated. However, Ig which target other regions of the RBD or in some instances the RBM itself can be broadly neutralising [23], meaning they maintain activity against SARS-CoV-2 VOCs and even other sarbecoviruses. Ig which target these regions can neutralise viral infection through blocking the interaction with ACE-2 or preventing conformational changes in S2 required for membrane fusion [24].

The NTD is also an important epitope, co-dominant with RBD as a target for B cells [25]. Several neutralising antibodies have been identified which target the same epitope in the NTD, termed the ‘NTD supersite’ [26]. However, this epitope is under selective pressure and mutations in this region dampen or eliminate neutralisation capacity of these Ig. In contrast, Ig targeting non-supersite NTD are less susceptible to mutations and retain neutralising capacity against VOCs [24]. Ig targeting the NTD can also retain neutralising capacity against both ‘open’ and ‘closed’ configurations of S [27].

Igs targeting S2 epitopes can neutralise viral entry by preventing this structural rearrangement and subsequent membrane fusion. Neutralising epitopes on S2 are more highly conserved than on S1, so are a more common binding site for broadly neutralising antibodies [25]. The S2 stem helix region and fusion peptide are conserved across betacoronaviruses [28,29], and targeting these regions blocks fusion of viral and host cell membranes, preventing viral entry. However, highly conserved S2 epitopes often fail to mediate robust neutralisation but can instead elicit Fc effector functions [30,31].

## 3. Humoral Immune Correlates of Protection against COVID-19

IgG can be divided into 4 subclasses, named in decreasing order of abundance in the plasma, IgG1, IgG2, IgG3 and IgG4. They have unique capacities for antigen binding, effector functions and complement activation [32]. IgG1 and IgG3 are typically produced in response to soluble protein antigens and make up the majority of the IgG response to COVID-19 infection and vaccination [33,34,35,36].

### 3.1. IgG Titres Post-Vaccination Are a CoP against COVID-19

A robust induction of anti-spike IgG following COVID-19 infection is associated with protection from severe disease or transmission [37]. Similarly, a strong correlation between anti-spike IgG titres and vaccine efficacy was found across platforms, including mRNA, adenoviral vector, protein subunit and inactivated virus vaccines in geographically diverse populations [38].

Serum or plasma is the most typical clinical sample used to test for circulating IgG, though self-collected capillary blood samples have also been used [39]. IgG can also cross out of the blood onto mucosal surfaces where they can be detected across various sample matrices including nasal swabs [40,41], saliva samples [42] or bronchoalveolar lavage [43].

As spike is the vaccine antigen, in addition to anti-spike IgG titres more specific epitopes including the RBD, and subunits 1 and 2 (S1 and S2) are routinely included as IgG that bind the RBD have a high correlation with viral neutralisation capacity. Nucleoprotein is not present in mRNA or subunit vaccinations, so the presence of IgG against nucleoprotein (anti-N) can be used to distinguish those with a previous or breakthrough SARS-CoV-2 infection depending on the context [44,45].

Enzyme-Linked Immunosorbent Assay (ELISA) is a standard technique for characterising IgG titres [46]. Binding of Ig to target antigens is quantified by enzyme-mediated colour-change reactions. Chemiluminescence immunoassays (CLIA) are a variation on this technique where the indicator is a luminescent molecule. Advantages over standard ELISA include wider dynamic range, high signal intensity, high specificity, reduced incubation time and multiplexing capacity [47,48]. Multiplexing capacity enables screening of different antigens or epitopes in parallel, as well as including IgG that target different SARS-CoV-2 variants on a single measurement plate. Both in-house [49,50,51] and commercial [52,53] high-throughput, multiplexing immunoassays have been developed for screening large cohorts, such as the MSD V-PLEX SARS-CoV-2 Panel 2 which was selected by Operation Warp Speed in the US as the standard binding assay platform across its Phase III clinical trials [54,55,56,57,58,59].

Surface Plasmon Resonance (SPR) is an alternative label-free approach to measuring protein-protein interactions, where interactions are quantified directly [60]. Capturing agents such as antibodies or antigens are immobilised on a thin metal layer, such as a gold microchip. Using a lateral flow approach, the sample solution is washed over the chip, facilitating protein-protein interactions. These interactions change the SPR angle, or the angle of minimum reflectivity, which is measured by SPR sensors as changes in reflected light intensity. This is measured in real-time, allowing for quantification of association and disassociation rates, as well as quantification of bound protein [61]. This allows SPR to capture binding of low affinity antibodies with fast dissociation rates which can be lost during ELISA-based assays due to the longer incubation periods [62]. This has been employed to screen anti-SARS-CoV-2 antibody levels with similar accuracy than ELISA, but at much lower cost due to the absence of the enzyme labelling and in a reduced time [63]. Multiplexing of IgA, IgM and IgG against the RBD is also possible [64].

Multiple research questions can be addressed with these assays including the amplitude and dynamics of the anti-spike response post-vaccination over time [65], the difference in peak response and waning between convalescent or vaccinated cohorts [66], between infection naïve and hybrid immune (those that have received vaccination and also experienced COVID-19 infection) cohorts [67], and between vaccine platforms [39]. Multiplex assays can evaluate the breadth of the antibody response across a panel of SARS-CoV-2 antigens or epitopes where IgG titres against S or RBD from different SARS-CoV-2 variants can be compared [68], determine relative immunity against different viral strains as well as monitor for immune escape. Alternatively, the capacity of a panel of Ig isotypes, or even IgG subclasses [36] to bind a single SARS-CoV-2 antigen can be measured using a multiplex assay. Although most existing assays in clinical trials measure IgG, the relevance of other classes of Ig (IgA and IgM) as biomarkers are discussed in Section 4.

### 3.2. Neutralisation Capacity Post-Vaccination Is a CoP against COVID-19

Along with anti-spike IgG titre, neutralisation titre (NT) is a commonly accepted CoP in COVID-19 vaccine trials [69]. Data from COVID-19 outbreaks [70,71] well as vaccine immunogenicity studies in non-human primates [72,73,74] have shown NTs to be a robust biomarker of protection against COVID-19 infection and severe disease.

Virus neutralisation assays are used to determine the NT of Ig present in a clinical specimen, which is the reciprocal dilution factor of a subject sample (plasma/serum) required to result in loss of inhibition of viral infection of target cells by a given percentage; 50% (NT50), 90% (NT90) etc. Although neutralisation assays are most frequently carried out on plasma/serum samples, they can be used to measure mucosal immunity, through saliva, nasopharyngeal swabs [41] or broncheoalveolar lavage [43].

Viral neutralisation assays do not distinguish the contributions of particular Ig isotypes or subclasses, but quantify the neutralisation capacity of all Ig present in the sample. However, when performed in parallel with binding assays, anti-S1 IgG titres and more specifically anti-RBD IgG titres are shown to correlate strongly with underlying NT [75,76].

#### 3.2.1. Live Virus Neutralisation Assays

Several methods are available to measure NTs experimentally (Figure 1). Live-virus assays use replication-competent SARS-CoV-2 clinical isolates, also referred to as “live-virus”. Plasma or serum samples are heat inactivated, to deactivate the complement pathway before being serially diluted and co-incubated with SARS-CoV-2. This facilitates binding of Ig to virion surface proteins. The antibody-virus mixture is then used to infect target cells in culture. These are typically Vero E6, an immortalised line originating from African Green Monkey kidney cells [77] or Vero E6/TMPRSS2 cells [78]. Other cellular models have also been used, including Caco-2 cells [79], originating from human colon epithelium, and A549 cells expressing ACE2 and TMPRSS2 [80] originating from human lung carcinoma.

Infection levels can be quantified through subjective observation of cytopathic effects (CPE), which includes counting plaques formed by virus-induced cell lysis in the cellular monolayer (Plaque Reduction Neutralisation Test, PRNT) or a 50% tissue culture infectious dose (TCID50) assay, or via objective immunodetection of viral foci or localised groups of infected cells (focus-reduction neutralisation test, FRNT). PRNT typically requires larger well sizes to facilitate visual plaque counting, while other assays, referred to as micro-neutralisation assays, have been developed specifically for 96-well plate formats, enabling higher-throughput sample screening.

While CPE can typically be assessed by eye [81], or with a light microscope [82,83], immunodetection of infected cells is quantified using microplate readers [84] or flow cytometry [85]. Alternatively, recombinant SARS-CoV-2 encoding fluorescent reporter proteins mNeonGreen or nanoluciferase (NLuc) allow infected cells to be observed directly using live-cell imagers [86] or by measuring luciferase intensity [87]. Another approach is using RT-qPCR to quantify virions released by infected cells (viral load) or cell-associated viral RNA, which is a measure of viral replication within cells [88,89]. Objective measurements of viral infection have advantages over CPE-based approaches as assessment of CPE can differ between observers.

The advantages of using live-virus for neutralisation studies is that the full range of SARS-CoV-2 surface antigens are available in their natural presentation for Ig-binding, and new or emerging viral variants can be incorporated into assays promptly once isolated. The main disadvantage of this approach is that as a Risk Group 3 pathogen, SARS-CoV-2 infection assays must be performed under Biosafety Level 3 containment, in certified Containment Level 3 laboratories with highly trained staff.

#### 3.2.2. Pseudovirus Neutralisation Assays

Pseudovirus neutralisation assays are an alternative to live-virus assays. Here a viral backbone from vesicular stomatitis virus (VSV), human immunodeficiency virus (HIV), or murine leukemia virus (MLV) is engineered to produce chimeric viruses, expressing full length spike protein from WT SARS-CoV-2 or viral variants as well as a reporter gene which expresses green fluorescent protein (GFP) or luciferase within infected cells. These pseudoviruses are typically replication-incompetent, meaning only a single replication cycle can occur [90,91].

The advantages of pseudovirus neutralisation assays are that they require only Biosafety Level 2 containment, making them accessible to a broader range of research institutions and a popular choice for high-throughput screens. A disadvantage of these assays is that only a single SARS-CoV-2 protein is included, so contributions of other antigenic binding sites to neutralisation are lost. Furthermore, the density or geometry of S on the surface of SARS-CoV-2 is not captured by S-expressing pseudoviruses, which may impact neutralisation.

#### 3.2.3. Implementation of Viral Neutralisation Tests in COVID-19 Clinical Trials

NT has been included in the assessment of vaccine immunogenicity as a primary or secondary endpoint of multiple COVID-19 vaccine trials. A range of pseudovirus and live-virus neutralisation assays with CPE, fluorescence and immunostaining endpoints have been utilised as outlined in Table 1. While these assays are all appropriate tools for assessing neutralising capacity, the range of assays used, in combination with inter-lab variability makes it difficult to directly compare results across independent studies. Indeed, in some cases, there has been more observed variation in NTs between different laboratories studying the same vaccine, than between different vaccines [92].

To facilitate comparison across studies and datasets, the World Health Organisation provided panels for the standardisation of laboratory COVID-19 immunological assays, which allows the reporting of Ig titres in Binding Antibody Units (BAU/mL) and neutralisation titres in International Units (IU/mL) [93]. However, even with this standardisation, significant variability remains. Geometric mean neutralisation titres reported for mRNA-1273 vaccine across six published estimates ranged from 247–1404 IU/mL, while the ChAdOx1 vaccine estimates ranged from 23–144 IU/mL [94]. Standardisation of assay selection and trial design would increase the utility of data obtained from these trials by facilitating cross-platform analysis of immunogenicity.

**Table 1:** Specific Immunoassays selected in Key COVID-19 Clinical Vaccine Trials.

Vaccine	Type	Phase	Trial ID	Regimen	Study Population	Location	Immunogenicity Endpoints	Humoral Immune Assays	Ref
BNT162b2 (Comirnaty), Pfizer/ BioNTech	Lipid nanoparticle encapsulated mRNA expressing pre-fusion stabilised, membrane anchored SARS-CoV-2 full-length S protein	III	NCT04368728	Primary	Infection naïve adults (>16 years)	USA, Argentina, Brazil, South Africa, Germany, Turkey	NA	NA	[95]
		I/II	NCT04368728	Primary	Infection naïve adults (18–55 or 65–85 years)	USA	Binding antibodies against WT Spike (RBD and S1) Neutralising antibodies against WT SARS-CoV-2 (NT50 and NT90)	RBD–binding or S1-binding IgG direct Luminex immunoassays FRNT-mNeonGreen	[16]
		I/II	NCT04380701	Primary	Infection naïve adults (19–55 years)	Germany	Binding antibodies against WT Spike (RBD and S1) Neutralising antibodies against WT SARS-CoV-2Neutralising antibodies against S mutants T cell responses	RBD–binding or S1-binding IgG direct Luminex immunoassays FRNT-mNeonGreen VSV-SARS-CoV-2 pseudovirus neutralisation assay	[96]
		III	NCT04955626	Monovalent vs. Bivalent booster	Older adults (>55 years)	USA	Neutralising antibodies against WT or Omicron BA.1, BA.2.75, BA. 4 and BA. 5 SARS-CoV-2	Fluorescent FRNT	[97]
Nuvaxovid (NVX-CoV2373), Novavax	Recombinant nanoparticle containing full-length spike plus matrix adjuvant	I/II	NCT04368988	Primary	Infection naïve adults	Australia, USA	Binding antibodies against spike Neutralising antibodies against WT SARS-CoV-2 T cell responses	S Total IgG ELISA CPE-based Micro-neutralisation assay	[17,98]
		III	2020-004123-16	Primary	Infection naïve adults (18–59 years)	UK	Binding antibodies against spike Neutralising antibodies against WT SARS-CoV-2 T cell responses	N IgG immunoassay S IgG ELISA Micro-neutralisation assay	[99]
		I/II	NCT04889209	Booster	Infection naïve, vaccinated adults (>18 years)	USA	Binding antibodies against WA-1-S-2P, Beta, Delta and Omicron spike Neutralising antibodies against WT and Omicron SARS-CoV-2 T cell responses	S IgG electrochemiluminescence immunoassay (MSD) Lentiviral pseudovirus neutralisation assay	[100]
ChAdOx1 (AZD1222), AstraZeneca	Replication-defective chimpanzee adenovirus-vectored vaccine expressing the full-length SARS- CoV-2 spike glycoprotein gene	I/II	NCT04324606	Primary	Infection naïve adults (18–55 years)	UK	Binding antibodies against spike and RBD Neutralising antibodies against WT SARS-CoV-2 (NT50 and NT80)	S Total IgG ELISA S and RBD Multiplex electrochemiluminescence immunoassay (MSD) PRNT FRNT	[101]
		III	NCT04516746	Primary	Infection naïve adults (>18 years)	USA, Chile, Peru	Binding antibodies against spike Neutralising antibodies against WT SARS-CoV-2	ELISA S, RBD and N multiplexed immunoassay (MSD) Live-virus neutralisation assay Pseudovirus neutralisation assay	[102]
		II/III	NCT044008383	Primary regime efficacy against B.1.1.7	Infection naïve adults (>18 years)	England, Wales, Scotland	Binding antibodies against spike and RBD Neutralising antibodies against WT or B.1.1.7 SARS-CoV-2	S Total IgG ELISA PRNT	[103]
		II/III	NCT04973449	Booster of variant updated vaccine (Beta)	Healthy COVID-19 vaccinated adults (>18 years)	UK, Poland	Binding antibodies against spike Neutralising antibodies against WT, Beta, Delta and Omicron SARS-CoV-2 T cell responses	Multiplex electrochemiluminescence immunoassay Pseudovirus neutralisation assay	[8]
CoronaVac, Sinovac	Inactivated whole virion	III	NCT04582344	Primary	Infection naïve adults (18–59 years)	Turkey	Binding antibodies against WT RBD Neutralising antibodies against WT SARS-CoV-2	RBD IgG and IgM chemiluminescence immunoassayCPE-based micro-neutralisation assay	[104]
		II	NCT04352608	Booster	Infection naïve adults (18–59 years)	China	Binding antibodies against WT Spike Neutralising antibodies against WT SARS-CoV-2	CPE-based micro-neutralisation assay	[105]
mRNA-1273 (Spikevax), Moderna	Lipid nanoparticle-encapsulated mRNA encoding the prefusion-stabilised full-length S protein	I	NCT04283461	Primary	Older adults (>56 years)	USA	Binding antibodies against S-2P and RBD Neutralising antibodies against WT SARS-CoV-2 (NT50 and NT80) T cell responses	S-2P-binding IgG ELISA nLuc neutralisation assay FRNT-mNeonGreen PRNT	[106]
		III	NCT04470727	Primary	Infection naïve adults (>18 years)	USA	NA	NA	[107,108]
		III	NCT04470427	Primary	Infection naïve adults (>18 years)	USA	Binding antibodies against WT Spike Neutralising antibodies	Electrochemiluminescence immunoassay (MSD) ELISA Pseudovirus neutralisation assay	[109]
		III	NCT05249829	Monovalent vs. bivalent boosters	COVID-19 vaccinated adults (>16 years)	UK	Neutralising antibodies against WT and Omicron BA.1	Pseudovirus neutralisation assay	[110]
JCOVDEN (Ad26.COV2.S), Jannsen	Non-replicating, recombinant human adenovirus type 26 viral vector encoding pre-fusion stabilised, membrane anchored SARS-CoV-2 full-length S protein	I/II	NCT04436276	Primary	Healthy adults (18–55 years)	USA	Binding antibodies against spike and RBD (WT and variants) Neutralising antibodies against WT SARS-CoV-2 and VOCs Fc effector functions T cell responses	ELISA Electrochemiluminescence immunoassay (MSD) Pseudovirus neutralisation assay nLuc neutralisation assay FcγR binding assay Cell-based ADCP, ADNP and ADCD assay	[111]
		I/II, III	NCT04505722	Primary	Healthy adults (>18 years)	Argentina, Brazil, Chile, Colombia, Mexico, Peru, South Africa, and USA	NA	NA	[112,113]
		III	NCT04614948	Booster	Infection naïve vaccinated adults (>18 years)	Belgium, Brazil, Columbia, France, Germany, the Philippines, South Africa, Spain, the UK, and USA	Binding antibodies against WT Spike	ELISA	[114]

#### 3.2.4. Surrogate Virus Neutralisation Test

As described above, cell-based viral neutralisation assays have inherent variability, require a minimum of Biosafety Level 2 handling, sterile working conditions, and assays can take a number of days to complete. The surrogate virus neutralisation test (sVNT) is an in vitro competitive binding assay used to measure the capacity of antibodies to block the interaction between recombinant RBD and recombinant human ACE2 (hACE2) receptor, either of which can be conjugated with a horse radish peroxidase (HRP) reporter. RBD is incubated with serum to allow RBD-targeting immunoglobulins to interact with target epitopes on the protein. The interaction between HRP-conjugated RBD to immobilised hACE2, or HRP-conjugated hACE2 to immobilised RBD can be measured as a reduction of HRP luminescence to indicate neutralisation [115,116].

The sVNT benefits from rapid turn-around of results, suitable for high-throughput screening and automation, higher reproducibility than cell-based assays, and can be performed under Biosafety Level 1 conditions. However, it does have disadvantages. The RBD harbours many neutralising epitopes, but neutralising antibodies can also bind sites across the S1 and S2 subunits of the spike protein (Box 1), which are not incorporated into this sVNT assay. While blocking ACE-2 binding is an important mechanism of viral neutralisation, other mechanisms, such as blocking viral-cell membrane fusion are also critical, and cannot be measured using this assay. Finally, as discussed above, soluble RBD may not accurately reflect the true post-translational structure of RBD within the spike region of a whole intact virion. SARS-CoV-2 spike has a complex and dynamic trimeric structure, which can be presented in an ‘up’ or ‘down’ conformation, and this influences the accessibility of epitopes to immunoglobulin binding [117].

sVNT has been used to study immune response and protection from breakthrough infection following a two-dose regime of BNT162b2 COVID-19 vaccine in adults [118] and in a paediatric cohort [119]. sVNT can be useful in instances where infectious virus or an appropriate cellular model is not available such as the work of Jia et al. evaluating whether SARS-CoV-2 infection and/or vaccination elicited cross-protective neutralising antibodies against other sarbecoviruses [120].

### 3.3. IgG Threshold Titres of Protective Immunity

Thresholds of protective immunity are valuable endpoints for clinical trials and can support clinical decision making when antibody levels above a pre-determined IgG threshold are indicative of protection. Following the publication of several Phase III COVID-19 vaccine clinical trials, both anti-spike IgG titre and NT were found to be CoP [121]. Quantifying IgG titres post-vaccination can be performed in a rapid, reproducible and high-throughput format. However these assays do not directly assess functional capacity. Neutralisation assays, in contrast, directly measure the functional capacity of Ig to block infection of cells, but can take several days to perform in Biosafety Level 2 or 3 facilities. Although discordance between IgG titres and NTs has been observed, particularly against Omicron variants [122,123,124], NTs typically correlate well with anti-spike IgG, particularly anti-S1 and anti-RBD.

As a result, studies have aimed to use the relationship between IgG, NT and clinical efficacy to define an IgG titre that would be predictive of robust neutralising capacity. PREVENT-19, the Phase III trial of the NVX-CoV2373 vaccine found an NT50 of 100 IU/mL corresponded to a vaccine efficacy against symptomatic COVID-19 of 81.7% [57] while the COVE Phase III trial of mRNA-1273 found an NT50 of 100 IU/mL corresponded to vaccine efficacy of 91% [55]. Lack of assay standardisation across clinical trials has led to a diverse range of NT being shown to correlate with vaccine effectiveness. Khoury et al. developed a vaccine-comparison model which could be used for immunobridging studies, using normalised neutralisation Geometric Mean Titres (GMTs) to determine non-inferiority or superiority margins a new vaccine would need to fall within to have sufficient expected efficacy. This model proposed a non-inferiority margin of 0.44-fold of the GMT in mRNA-1273 vaccines, or 0.54-fold the GMT in BNT162b2 vaccines that would predict vaccine efficacy of >80%, while a superiority margin of 2.6-fold the GMT was required for ChAdOx1 vaccinees [94].

IgG titres have also been shown to correlate with vaccine efficacy, with one study showing a protective threshold of 154 BAU/mL to be correlated with protection against WT SARS-CoV-2 infection across 6 vaccination regimes, though this increased up to 490 BAU/mL to retain protection against the Delta variant [125]. Kenny et al. identified an anti-RBD titre of 456 BAU/mL in subjects with confirmed prior SARS-CoV-2 infection was highly predictive of an NT50 > 1000 IU/mL against WT SARS-CoV-2 using a live-virus neutralisation assay, with this RBD threshold retaining similar protection when tested in hybrid-immunity cohorts and against immune-escape variants [76]. An anti-RBD IgG titre below this threshold could predict poor neutralising capacity against the Beta and Omicron BA.5 variants of concern (VOC) in those with diverse COVID-19 infection and vaccination histories. A similar anti-RBD IgG threshold of 506 BAU/mL correlated with a vaccine efficacy (ChAdOx1) of 80% against infection with SARS-CoV-2 Alpha variant [58].

Having a defined threshold of protective immunity would also allow identification of populations who would benefit from additional booster doses, and support the implementation of an efficient booster schedule [126]. It would also allow better correlation between vaccine platforms and analysis of their performance against SARS-CoV-2 VOCs. However, a universal threshold is unlikely to be accepted unless trial protocols are standardised to reduce the impact of assay variation on output. The use of International Units in reporting of results is essential to allow comparisons between studies. Furthermore, even with these in place, the definition of a protective threshold must be continually adapted in line with the introduction of new viral variants and the shifting immunological landscape in response to repeat vaccination and multi-valent vaccines.

## 4. Contribution of Other Humoral Immune Biomarkers to COVID-19 Vaccine Efficacy

While neutralising capacity is the most extensively studied circulating Ig effector function when it comes to protection against COVID-19 transmission and severe disease, mucosal immunity, Fc-effector functions, and the generation of long-lived plasma and memory B cells also contribute to overall vaccine effectiveness. Though outside the scope of this review, T cell mediated responses have a critical role which has been reviewed elsewhere [127,128,129]. Furthermore, VOCs such as Omicron have shown considerable immune escape from vaccine-induced virus neutralisation prior to introduction of bivalent vaccines, but hospitalisation and death were still reduced following vaccination [130]. The COVE study also estimated only about two-thirds of mRNA-1273 vaccine efficacy was mediated by neutralising antibodies at day 29 post vaccination [55]. Hence, it is important to characterise other determinants of vaccine effectiveness.

### 4.1. Fc-Effector Functions

While neutralisation is important in preventing cells from becoming infected, Fc effector functions are critical for the elimination of infected cells and resolution of infection [131]. Antibody-dependent cellular cytotoxicity (ADCC) and antibody-dependent cellular phagocytosis (ADCP) are mediated by Fc domains of Ig which, once bound to their cognate antigen, are recognised by specific Fc receptors (FcRs) expressed at the surface of effector cells. ADCC effectors include natural killer (NK) cells, monocytes, macrophages, neutrophils, eosinophiles and dendritic cells. For those, activation of FcγRIIIa triggers release of cytokines such as IFN-y that lead to ADCC-mediated killing of the infected target cell, though other FcγRs may also have a role [132]. ADCP effector cells include monocytes, macrophages, neutrophiles or dendritic cells, which, following engagement of their FcγRIIa, mediate the phagocytosis of infected target cells [133]. Antibody-dependent complement deposition (ADCD) is mediated not through effector cells but through the complement system, a network of soluble proteins in the blood. The classical complement activation pathway is activated by interaction of the Fc domain of IgM or IgG with the C1q subunit of C1. This initiates an enzymatic cascade ultimately leading to formation of a Membrane Attack Complex (MAC) which forms a membrane pore, facilitating the lysis of the target cell [134].

Fc effector functions are independent of Ig neutralising capacity. They can be observed prior to the development of a robust neutralising antibody response [135], and after neutralising antibodies have waned [136]. Of note, Ig that elicit Fc-effector functions are not restricted to binding the RBD region and plasma from COVID-19 vaccinated individuals retains Fc-effector functions even when RBD-targeting antibodies are removed [135]. The S2 domain is highly conserved across SARS-CoV-2 VOCs as well as other beta coronaviruses so Ig targeting this site could provide improved cross-protection [137,138].

ADCP, ADCC and ADCD are all induced by COVID-19 infection and vaccination. While the breadth of the IgG response against spike is higher in mRNA vaccinated (BNT162b2 or mRNA-1273) than convalescent cohorts, the breadth and potency of FcR binding is higher still, suggesting that vaccination not only boost SARS-CoV-2 specific IgG production with regards to titre, but also with more breadth of functionality [139]. Interestingly, ADCD is the only effector function to be greater in convalescent cohorts. This could be attributed to IgM titres. IgM is a strong inducer of the classical complement pathway, and is less common in vaccinated cohorts than following infection [139] as explored in Section 4.3. While ADCD declines after 4-5 months, ACDP could contribute to longer-term protection from severe disease [140].

#### 4.1.1. Evaluating Fc-Effector Functions in Clinical Trials

##### Fc Effector Cell-Based Assays

**Figure 2:**
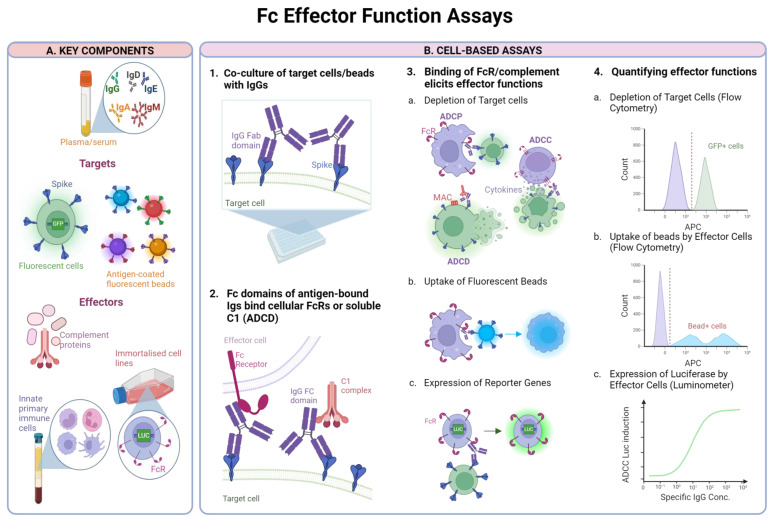
Fc Effector Function Assays. (**A**) Assays require plasma or serum, target cells or beads expressing or coated with SARS-CoV-2 antigens (spike), and effectors including complement proteins, primary innate immune cells or genetically modified cell lines with luciferase (luc) reporter gene. (**B1**) Target cells or beads are incubated with Ig facilitating the binding of specific Ig Fab-domains to SARS-CoV-2 antigens on the cell surface. (**B2**) Antigen-bound Ig can bind FcRs on effector cells or bind soluble complement via their Fc domains. (**B3**) This results in (**a**) elimination of target cells through Antibody-Dependent Cellular Phagocytosis (ADCP) or Antibody-Dependent Cellular Cytotoxicity (ADCC) or Antibody-Dependent Complement Deposition (ADCD), (**b**) uptake of target beads by ADCP or (**c**) expression of luciferase by effector cells. (**B4**) Flow cytometry can be used to measure (**a**) reduction of fluorescent target cells, or (**b**) uptake of fluorescent beads by effector cells. (**c**) Luminometry or spectrophotometry can be used to measure expression of luciferase by effector cells. Created with Biorender.com (accessed on 19 September 2024).

ADCC, ADCP and ADCD can be examined experimentally by quantifying the killing of target cells infected in vitro with SARS-CoV-2 or expressing SARS-CoV-2 antigens following their incubation with serum/plasma from convalescent or vaccinated individuals and either effector cells or complement depending on the function being measured (Figure 2). There, Ig can detect their cognate epitope via their Fab domain while their Fc domain can interact with FcRs present on effector cells or activate complement leading to death of the target cell through cytotoxicity, phagocytosis or opsinisation. Reduction in target cells over time is a measure of effector function [141]. Target cells can be transduced with GFP so depletion of GFP positive cells can be easily monitored using flow cytometry.

To replace primary effector cells, ADCC and ADCP reporter bioassays can include reporter effector cell lines, such as genetically modified Jurkat T cells expressing FcγRIIIa (mediates ADCC) or FcγRIIa (mediates ADCP) and an NFAT-RE luciferase reporter gene. Interaction between antigen-bound Ig and FcRs of the effector cells activates the nuclear factor of activated T cells (NFAT) and drives expression of the luciferase reporter gene which can be quantified as a proxy for ADCC or ADCP activity, depending on the receptor being expressed [142].

In the case of ADCP, the target cells can be replaced with fluorescent beads, coated with the antigen of interest. Multiple bead colours allows for multiplexing of several antigens in a single well [143]. Here, beads are incubated with the plasma/serum samples before incubation with a human monocyte cell line THP-1 [144] or with neutrophils, isolated from primary peripheral blood mononuclear cells (PBMCs) [145], to measure to measure ADCP or antibody-dependent neutrophil phagocytosis (ADNP) respectively.

##### Fc Effector In Vitro Binding Assay

Distinct Ig isotypes have specific receptors on the surface of immune cells including B cells, macrophages, natural killer (NK) cells and mast cells. These are Fc gamma receptors (FcγRs) for IgG, FcαRI for IgA, FcμR for IgM and Fcα/μR for IgA and IgM. As this physical interaction of antigen-bound Ig with the complement pathway or FcRs is the first and essential step of triggering Fc-mediated functions, measuring receptor binding capacity can predict the functional capacity of Ig.

In place of cell-based assays, in vitro Fc effector assays have been developed which quantify binding of antigen-bound Fc to soluble FcRs or complement as a proxy for Fc effector functions. These assays utilise magnetic beads coated with the antigen of interest to capture the specific antibodies in biological samples. The antigen-antibody complex is then incubated with recombinant biotinylated FcRs [146,147] or with lyophilised guinea pig complement to measure ADCD [148]. Fluorescently tagged antibodies against either total IgG or specific IgG subclasses are then added which can be analysed using flow cytometry. Fluorescently coded magnetic beads allows up to 500 antigens to be multiplexed using an array reader [146]. Such assays would therefore be suitable for large screening studies.

#### 4.1.2. COVID-19 Vaccination Induces Potent Fc Effector Functions

Key relevant studies focused on FcR binding and effector function of vaccine-induced Ig versus those produced in response to COVID-19 infection, show a hierarchy of Fc effector responses, with hybrid immunity being the most robust, and convalescence being the weakest.

mRNA vaccinated individuals were shown to have improved FcγR binding compared to individuals post-infection and this improvement was greater than the increase in IgG titres, suggesting post-vaccination IgG were more functional. Vaccinated plasma was also shown to bind FcγRIIa and FcγRIIIa, associated with ADCP and ADCC respectively, with greater affinity than convalescent plasma [149]. Functional assays supported this finding, showing that ADCP and ADCC, but not ADCD were increased in a vaccinated cohort [139]. Those with hybrid immunity had a greater increase in FcR binding compared to infection-naïve, vaccinated individuals, although the gap reduced with repeat vaccination. Hybrid-immunity was also associated with enhanced binding to FcγRIIa and FcγRIIIa, which may enable those with hybrid immunity to kill infected cells more efficiently [137].

##### FcR Binding Antibodies Are More Cross-Reactive than Neutralising Antibodies against VOCs

Fc-mediated functions are independent of Ig neutralising capacity. Both should be evaluated to more fully characterise the full-spectrum of vaccine-induced Ig response. Breadth of anti-spike IgG is increased in vaccinated cohorts (mRNA vaccines) compared to convalescent individuals, which is associated with cross-protection against VOCs and other coronaviruses [139]. Indeed, plasma from vaccinated individuals was more efficient at binding RBDs across a panel of VOCs. While immune-escape was observed, the decrease in FcγR binding antibodies produced against Omicron BA.2 compared to WT SARS-CoV-2 was significantly less than the decrease in neutralising antibodies (3.1–3.7-fold vs. 26-fold reduction), suggesting FcγR binding antibodies are more robust against viral mutations than neutralising antibodies [149]. A clinical trial of mRNA-1273 vaccine that aimed to define the humoral vaccine response in children, examined Ig binding to FcRs as well as capacity to carry out specific effector functions including ADCD and ADCP [150]. They observed that following vaccination with an adult dose of mRNA-1273, IgG specific to the Omicron RBD was substantially reduced compared to those against WT RBD and those of other VOCs. Neutralisation capacity, which is primarily mediated by RBD-specific antibodies, was also substantially reduced. In contrast, Omicron spike-specific IgG were well conserved, and the children had minimal loss of Fc-binding antibodies targeting the Omicron spike protein. These studies suggest that Fc-binding antibodies are more well conserved than neutralising antibodies against SARS-CoV-2 VOCs, perhaps due to the greater breadth of spike-specific Fc-binding IgG which renders them less sensitive to mutations.

#### 4.1.3. IgG Subtypes Have Diverse Fc Effector Functions

##### Fc Effector Functions and IgG Subtypes

All IgG subclasses can bind and neutralise viruses, though IgG1 and IgG3 are the most potent triggers of Fc-mediated effector mechanisms, with IgG2 and IgG4 showing reduced affinity for FcγRs [32,151]. IgG4 is often considered as anti-inflammatory. IgG4 binding to most FcγRs is impaired, resulting in reduced ADCC and ADCP activity. In contrast, binding to FcγRIIb, an inhibitory Fc receptor, found on a range of immune cells including B cells, monocytes and macrophages, is increased compared to the other subclasses, which skews IgG4 activity towards inactivating effector functions. IgG4 is also a poor activator of complement, with minimal capacity to induce ADCD through the classical route [152].

##### Distinct IgG Subclass Dynamics after mRNA Versus Adenoviral Vaccination

Several studies have measured the contribution of specific IgG subclasses to the total IgG response post-COVID-19 vaccination, with either mRNA or adenovirus-vector vaccines, and have noted significant differences in induction of IgG4 after repeat vaccination. Early post infection, there is a dominant IgG1 response, followed by IgG3 [33,34,35] similar to what is observed in convalescence. Interestingly, in infection naïve individuals, mRNA vaccination (BNT162b2 or mRNA-1273) induces a late IgG4 response, several months post-vaccination, which was not seen in those vaccinated with adenoviral vectors [153]. 7 months following two doses of mRNA vaccination (BNT162b2), 50% of those tested had a measurable IgG4 response, compared to 2% who received the ChAdOx1 (adenoviral vector) vaccine. A third dose of BNT162b2 increased the titres of IgG4, which accounted for up to 80% of total anti-spike IgG in those who had subsequent breakthrough infections [154]. Following booster doses of mRNA vaccines (BNT162b2 or mRNA-1273), IgG4 overtakes IgG1 as the dominant IgG. Furthermore those who received ChAdOx-1 as a primary series but an mRNA booster also had a rise in IgG4 titres after six months [155]. A fourth study has also observed a rise in IgG4 titres following the booster dose of BNT162b2 in a previously infection naïve cohort, and they showed no decline in the IgG4 titres over 9 months follow up [156]. The mechanisms of IgG4 induction by mRNA vaccination have not been elucidated but may be associated with prolonged exposure of germinal centres to vaccine mRNA which has been detected 60 days post-vaccination [157].

##### IgG4 and Fc Effector Functions

The clinical significance of this induction of IgG4 remains poorly understood. Sera taken after three mRNA vaccinations had significantly lower phagocytosis capacity than sera from the same donors after 2 vaccinations [154]. Engagement of IgG4 with FcγR2a results in reduced activation of the receptor which is associated with reduced ADCP. ADCD capacity was also significantly reduced after 3 vaccinations [154] and IgG4 is known to poorly activate the classical complement pathway [152].

Inclusion of IgG subclasses in clinical studies could answer questions remaining surrounding the choice of vaccine vectors, the impact of repeat boosters on long-term immunity, and the role of neutralisation versus effector functions in controlling viral transmission and replication.

While IgG titres are strongly induced by COVID-19 infection and vaccination, and correlate well with neutralising and effector functions, a broad repertoire of Ig, including IgA and IgM is associated with higher neutralisation capacity [158], highlighting a role for inclusion of these Ig when measuring the humoral response [159].

### 4.2. IgA Titres

IgA is the most prevalent secreted Ig on mucosal surfaces, where it contributes to immune exclusion through agglutination of pathogens preventing their adsorption. As well as neutralising SARS-CoV-2, IgA can mediate multiple effector functions, including phagocytosis, superoxide generation, cytokine release or neutrophil extracellular traps (NETs) [160]. As with IgG, both IgA and IgM can be measured from serum or plasma samples using standard binding antibody assays, such as ELISA or chemiluminescence [161,162,163,164].

Dimeric IgA associated with mucosal surfaces has a higher affinity for spike and more potent neutralisation capacity than monomeric IgA, which is the predominant circulating form [165]. Neutralisation capacity of nasal secretions is associated with anti-RBD IgA titres in nasal secretions but not anti-RBD IgG highlighting the important role of IgA in mucosal immunity [41].

Mucosal immunity is highly effective at preventing infection and limiting transmission by blocking SARS-CoV-2 replication at the site of entry and thus preventing disease progression [166]. While COVID-19 vaccines are highly successful at limiting severe disease and death, breakthrough infections are common, accounting for 80% of SARS-CoV-2 infections and re-infections in the Omicron era [167,168,169]. This could be partly due to poor induction of mucosal immunity elicited by peripheral, intra-muscular vaccination, while SARS-CoV-2 infection can induce mucosal immunity due to direct exposure of the URT to SARS-CoV-2 antigens [166]. Only a small percentage of vaccinated individuals maintain a secretory IgA response for up to 6 months after 2 doses of mRNA vaccination, which is associated with reduced risk of breakthrough infections [170]. In saliva, the presence of IgA was undetectable following a 2-dose BNT162b2 regime in naïve individuals [171]. Following a booster, salivary IgA was detected in those with high titres of circulating IgA but this could reflect circulating IgAs that leaked from the serum rather than true mucosal IgA [172]. However, in those with a prior infection history, vaccination can induce a secretory IgA response [42,43,170]. In hybrid immune individuals, a robust mucosal IgA response but not IgG is associated with reduced risk of breakthrough infections following exposure to the Omicron VOC [173].

Intranasal vaccines are already under development [174,175,176,177,178]. These deliver the same SARS-CoV-2 Spike antigens or live-attenuated virus as intra-muscular vaccines but via an intra-nasal spray, which could induce a stronger mucosal immune response because the route of administration more closely resembles the natural route of most SARS-CoV-2 infection. These vaccines offer the potential of providing sterilising immunity, resulting from complete neutralisation of SARS-CoV-2 at the site of entry and thus eliminating transmission [160].

### 4.3. IgM Titres

IgM have multimeric structures, allowing them to bind antigens with high avidity, neutralise and aggregate virions and present them to secondary lymphatic organs, accelerating the induction of the adaptive immune response. IgM can bind FcRs triggering phagocytosis by macrophages and B cells, which contributes to antibody production and a robust germinal centre response [179] and facilitates presentation of antigens to T helper cells [180].

Typically IgM is the first Ig produced in response to infection so is useful as a biomarker of acute or recent infection. However, with SARS-CoV-2 infections, IgA and IgG are often produced before or with IgM, which is more typical of a memory response [181]. IgG cross-reactivity between SARS-CoV-2 spike protein and that of other human coronaviruses could explain this, whereby early IgG are memory derived and directed to more conserved spike epitopes, while IgM is naïve-derived and is therefore more specific to SARS-CoV-2 spike [164]. In the first week post-infection, neutralisation titre correlates most strongly with anti-spike IgM, followed by anti-spike IgG. [75]

Following COVID-19 vaccination, IgM induction is more common in SARS-CoV-2 infection naïve individuals. Most develop a non-canonical response, where IgM is either absent, or appears after the induction of IgG [162]. Those who have a canonical response, where IgM appears with or before IgG have a more efficient response, characterised by higher neutralisation capacity and increased duration and titre of anti-spike IgG antibodies. Thought the kinetics of IgM are not associated with neutralisation capacity in those with a prior history of infection [162,163], in infection-naïve individuals, a non-canonical IgM response was associated with breakthrough infections after completing a three-dose vaccine regime, [163].

For infection-naïve individuals, vaccine-induced protection from infection is not mediated directly by IgM, but rather the early induction of IgM in relation to IgG indicates a robust immune response, and this cannot be inferred without measuring both IgM and IgG at an early timepoint post-vaccination. Including an anti-spike IgM measurement early-on post-vaccination could identify individuals less likely to develop a robust humoral immune response to COVID-19 vaccination.

## 5. Memory B Cells as a Biomarker of Long-Term Vaccine-Induced Immunity

While evaluating antibody responses is critical in monitoring the immediate protective capacity of SARS-CoV-2 vaccines, parallel exploration of SARS-CoV-2 B cell responses is imperative for comprehending the serological response. Investigating memory B cell formation, maturation, and persistence answers crucial questions regarding humoral immune durability induced by vaccination, recall capacity for future vaccines and infection, and the breadth of their repertoire against VOCs.

### 5.1. B Cell Activation and Differentiation in Response to SARS-CoV-2

Production within the bone marrow generates a diverse pool of immature B cells, which subsequently migrate to the blood, mucosal surfaces, and lymphoid tissues as mature B cells. Although mature, these B cells are still antigen naïve. The stimulation of naïve B cells by SARS-CoV-2 antigen presentation and binding causes B cell differentiation into SARS-CoV-2 specific memory B cells, or plasma or antibody secreting cells (ASC). While the intricate cellular and molecular mechanisms underlying the generation and differentiation of B cells fall outside the scope of this review, a recent review by Inoue et al. offers detailed insights into these processes [182].

### 5.2. Challenges in Working with Memory B Cells

Measuring B cell responses starts with the isolation and/or cryopreservation of Peripheral Blood Mononuclear Cells (PBMCs) or B cells. Of note, cryopreservation can impact assay performance, significantly reducing cell viability in frozen samples viability [183,184]. Most studies focus on systemic immunity and circulating memory B cells, which constitute only a fraction of the total memory B cell pool. Memory B cells are more abundant and diverse in lymphoid tissues such as lymph nodes, spleen, tonsils, Peyer’s patches and the bone marrow. In the context of respiratory infections like SARS-CoV-2, mucosal-associated lymphoid tissue (MALT) in the upper respiratory tract (URT) and lower respiratory tract (LRT), as well as non-lymphoid barrier tissues like the lungs are particularly relevant. These sites harbour stable populations of tissue-resident memory B cells. Research on local mucosal immunity to SARS-CoV-2 is hindered by difficulties in accessing the respiratory sites, especially the lower airways, and the low yield of B cells obtained from these areas. Despite their low numbers in circulating blood, these cells still reflect the diversity of the extensive pool of memory B cells distributed throughout the body [185,186,187].

### 5.3. B Cell ELISpot (Enzyme-Linked ImmunoSpot) Assay for B Cell Response Assessment

**Figure 3:**
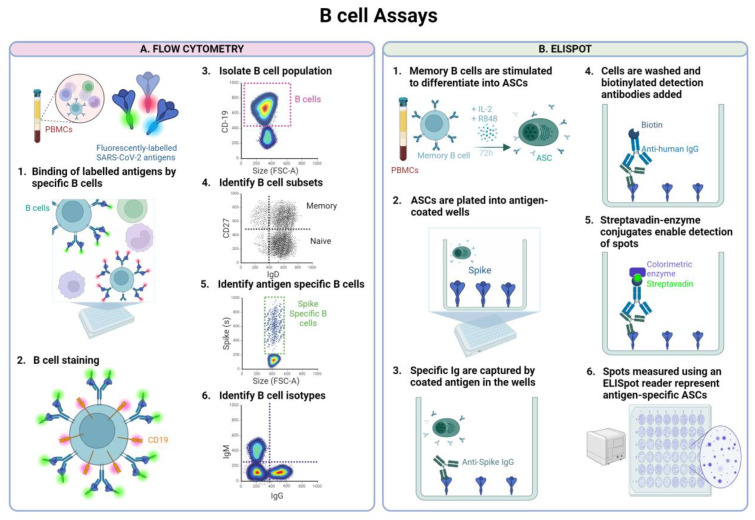
B cell Assays. (**A**) Peripheral Blood Mononuclear Cells (PBMCs) and fluorescently labelled SARS-CoV-2 antigens (spike). (**A1**) SARS-CoV-2 antigens are incubated with PBMCs and are captured by antigen-specific B cells. (**A2**) B cells are stained with fluorescent antibodies against B cell markers e.g., CD19 (**A3**,**A4**) B cells and B cell subsets can be identified via flow cytometry using B cell markers. (**A5**) B cells bound to the antigen of interest can be identified using the fluorescently-tagged antigens. (**A6**) B cell isotypes can be identified using fluorescently-tagged antibodies against specific Ig. Panel (**B**). (**B1**) Antibody Secreting Cells (ASC) can be isolated directly from PBMCs or memory B cells can be stimulated for 72 h to trigger differentiation into ASC. (**B2**) ASC are added to a plate coated with SARS-CoV-2 antigen (spike). (**B3**) Ig are continuously released by ASC. Spike-specific Ig bind surface-bound antigen local to the ASC. (**B4**) Cells and unbound Ig are removed by washing. Biotinylated anti-human IgG antibodies are added to detect specific-IgG bound to the surface antigen. (**B5**) Enzyme-labelled streptavidin is added to the well and is captured by the biotinylated detection antibodies. (**B6**) A substrate is added which forms a coloured insoluble precipitate when catalysed by the antibody-bound enzyme. Each antigen-specific ASC forms a single spot which can be read with an automated spot reader. Created with BioRender.com (accessed on 19 September 2024).

Measuring B cell response to SARS-CoV-2 vaccination or infection is generally performed through an Enzyme-Linked Immunospot (ELISpot) assay or flow cytometry (Figure 3). The B cell ELISpot assay pioneered by Czerkinsky in 1983 [188], is a highly sensitive method to measure the function and the frequency of cells secreting antibodies against specific antigens such as SARS-CoV-2 spike or RBD. While ASCs can be directly tested, memory B cells, because of their quiescent nature, require to be stimulated ex vivo, typically with IL-2 and R488, to differentiate into ASCs followed by plating. Subsequently, secreted specific antibodies are captured by SARS-CoV-2 antigens such as spike or RBD coated on a membrane, with each spot representing antibody secretion from an individual B cell. This is compared to the total Ig to determine the percentage of SARS-CoV-2 specific memory B cells. Following the same principle but in a multiplex format, the FluoroSpot B cell assay uses different fluorophores allowing for the detection and analysis of multiple SARS-CoV-2 antigens, Ig isotypes, or subclasses in a single well. A notable advantage of ELISpot assay is its sensitivity, capable of detecting very low frequencies of antigen-specific memory B cells (0.0002% total cells), requiring less cells than flow cytometry with 2 million PBMC recommended in protocols [189].

### 5.4. Flow Cytometry Technique for B Cell Response Assessment

Although ELISpot retrieves the frequency of cells that are secreting the SARS-CoV-2 Ig it does not provide information on the phenotype of responding cells. Much of SARS-CoV-2 vaccine research to date has focused on evaluation of B cell responses through flow cytometry which can enumerate antigen-specific B cells and antibody isotype while also facilitating the detailed characterisation of B cell subsets through expression of co-receptors or lineage markers [190]. The assessment of SARS-CoV-2 specific B cell responses through flow cytometry begins with preparing antigen probes with fluorescent labels designed to bind to SARS-CoV-2 antigen specific B cells. As with ELISpot, the antigens can be interchanged and updated in line with VOC specific antigens. PBMCs are subsequently stained with these SARS-CoV-2 antigen probes and B cell surface markers using fluorophore-conjugated antibodies to identify the B cell population of interest. In addition to surface marker staining, cells can also be permeabilised to allow for intracellular staining of cytokines for example antibodies targeting cytokines associated with B cell function, such as IL-10 or IL-21. Gating strategies are focused on identifying the subpopulations of interest. Notably, a significant limitation of flow cytometry is the requirement of higher numbers of PBMCs than ELISpot with between 5 to 10 million PBMCs recommended [191] to maintain sensitivity of the assay given the low frequency of antigen-specific B cells in peripheral blood [192]. Important to note while interpreting B cell response studies is the lack of a standardised international unit for B cell quantification, introducing subjectivity in reporting with both assessment techniques.

### 5.5. Persistence and Recall Capacity of Memory B Cells and Their Dynamic Responses to SARS-CoV-2

Memory B cells are pivotal in the immune system’s rapid and enhanced response to pathogen upon re-exposure. Recent studies on SARS-CoV-2 have provided critical insights into the B cell dynamics and their importance in sustained immunity.

Cui et al. detected spike-specific and RBD-specific circulating memory B cells in previously infected individuals using flow cytometry [193]. This correlated with accelerated SARS-CoV-2 spike-specific IgG production observed after the first vaccine dose compared to the infection-naïve group, whose IgG response increased only after the second dose.

Liu et al. observed that while neutralising antibodies declined over time, spike-specific and RBD-specific memory B cell responses persisted at 6 months post primary vaccinationas shown by B cell ELISpot assays [194]. Importantly, booster vaccination significantly elevated, both memory B cell responses and neutralising antibodies [194].

Ciabattini et al. conducted a 9 months longitudinal study post-primary vaccination series [195]. Using Flow cytometry, they observed increases in IgG+ and IgM+ resting memory B cells at 9 months, while spike-specific plasmablasts, including IgA+ and IgG+ subsets, peaked at month 3, before declining to minimal levels by 9 months, mirroring the circulating spike-specific IgA and IgG serological response. Despite declining circulating Ig, ex vivo stimulation and ELISpot assays revealed that spike-specific memory B cells remained functional [193].

Byrne et al. showed that RBD-specific memory B cells could be used as a CoP against COVID-19 infection. Lower frequencies of these cells were found in individuals who experienced breakthrough infections within 10 months of a third dose COVID-19 vaccine, compared to those who did not [196]. Anti-RBD IgG titres, by contrast were not significantly different between the groups, emphasizing the importance of assessing memory B cells responses in addition to antibody levels.

### 5.6. Memory B Cells Role in SARS-CoV-2 Hybrid Immunity

SARS-CoV-2 hybrid immunity provides more durable efficient protection against infection compared to natural infection or vaccination alone [197], resulting in antibodies with higher binding affinity and increased neutralising potency [198].

Goel et al. showed that prior SARS-CoV-2 infection enhances immunogenicity of a single vaccine in convalescent individuals, producing full spike-specific and RBD-specific memory B cell responses comparable to two doses of mRNA vaccine in infection-naïve individuals [199]. Pre-vaccination infection-derived spike S1 specific memory B cells predict post-vaccination B cell response, with significantly elevated counts of S1-specific IgG+ ASCs after vaccination in those with hybrid immunity [200].

Mitsi et al. demonstrated heightened SARS-CoV-2 specific memory B cell response in lung mucosa of individuals with hybrid immunity [201]. Spike-specific memory B cell responses were detectable only among those with hybrid immunity, and correlated with broncheoalveolar lavage (BAL) anti-spike IgG titres. Within this group, the frequency of S-specific memory B cells was notably higher in BAL samples compared to paired blood samples. While lung mucosa memory B cells were mainly class-switched, the paired blood samples had a significantly increased proportion of unswitched memory B cells [201]. This suggests that a long-lived, airway-compartmentalised B cell reservoir capable of producing a large repertoire of antibodies in the lung mucosa may confer better primary protection from infection.

Hybrid immunity increases RBD-specific memory B cells by recruiting new B cell clones into the memory pool and expanding persistent, clonally diverse, affinity matured B cell lineages expressing broad and potent antibodies through extensive somatic hypermutation [202,203]. This results in higher potency and breadth of neutralising antibodies against VOC compared to vaccination or infection alone [202,204].

Using multi-parameters flow cytometry and combinatorial gating strategy to analyse memory B cells, Quandt et al. analysed memory B cell subsets recognising variant-specific and shared epitopes outlining the breath and specificity of memory B cell responses following breakthrough Omicron BA.1 infection in individuals vaccinated with BNT162b2 (Pfizer-BioNTech, New York, NY, USA). This robust recall response expanded memory B cells against conserved epitopes rather than inducing de novo BA.1-specific B cell responses [205].

Understanding hybrid immunity humoral responses including memory B cell dynamics and plasticity is crucial for ensuring comprehensive protection and resilience against the evolving landscape of COVID-19 and its variants.

### 5.7. Role of Long-Lived Plasma Cells in SARS-CoV-2 Vaccine Immune Durability

Long-lived plasma cells (LLPC) provide durable immune protection following vaccination by continuously secreting antibodies, offering immediate protection upon re-exposure to the virus. Most LLPCs migrate to the bone while some localise to specific organs providing localised protection.

Research shows that COVID-19 vaccination induces spike-S1 and RBD-specific bone marrow LLPCs [206]. Using multi- targeted flow cytometry, they identified that about 20% of the SARS-CoV-2 spike-specific plasma cell population exhibited a B cells phenotype linked to increased longevity [206]. While limited data correlates LLPC presence with longterm circulating antibodies, their induction by vaccination is important for durable immunity given their ability to continuously produce antibodies without further activation.

LLPCs migration to respiratory mucosa following vaccination is crucial for localised primary protection. Detectable anti-spike IgG and IgA in BAL fluid of vaccinated individuals without prior infection supports the role of LPPCs in mucosal immunity [201]. Enhancing LLPC induction and migration could improve vaccine efficacy and long-term immunity against SARS-CoV-2.

While evaluating antibody levels and functions offers insights into immediate protection, understanding long -lived B cell immunity requires functional B cell assays. These assays offer a broader perspective of the immune response, including recall responses, memory pool replenishment, hybrid immunity and repertoire diversity.

## 6. Conclusions

The COVID-19 pandemic and global vaccination efforts has provided valuable insights for future vaccination development. Early clinical trials established assessed vaccine efficacy and CoPs for regulatory and clinical decision making [95,107,207]. For COVID-19, neutralisation titers and anti-spike IgG titres were quickly identified as CoPs [121] forming the basis for several immunobridging trials [4,5,7,8,9]. While CoPs can only be established following extensive clinical evaluation, establishing specific CoPs early will be essential to streamline clinical development.

The COVID-19 pandemic prompted unprecedented collaboration between industry and academia, leading to high throughput platforms for quantification of IgG titres and viral neutralisation [52,53,81,84,85,88,89,90,91], which have been promptly customised to emerging VOCs. While future pathogens will need to be characterised and specific biomarkers identified, these tools provide a framework for assessing B cell and antibody responses to viral pathogens, adaptable to new pathogens, as demonstrated with emerging variants of concern.

Challenges remain in defining CoPs. Population studies must be carried out to account for the diverse nature of vaccine responses, which can limit reproducibility and application of pre-defined thresholds across diverse cohorts. Differences in immune responses have been observed between hybrid-immune versus infection-naïve cohorts at the levels of IgG titres [67], mucosal immunity [42,43,170] and Fc effector functions [137]. Similarly, immune responses differ among different age groups and immunosuppressed individuals. Thus, vaccine response and effectiveness should be evaluated independently in higher risk populations.

Further research is also needed to understand the contribution of mucosal immunity in preventing infection [170]. COVID-19 vaccinations have been effective at reducing severe disease and death but less so at preventing transmission. Since the upper airway is the primary site of entry of SARS-CoV-2, understanding how to generate both mucosal and systemic immunity through vaccination could inform the design of future vaccine strategies against respiratory viruses, such as the development of intra-nasal formulations [177,178].

Finally, we highlighted the crucial role of memory B cells in long term protective immunity. While circulating Ig peak and wane after vaccination, long lived memory B cells can mount a rapid response upon re-exposure to the antigen. Comprehensive immune monitoring should include B cell responses, as the number of boosters, dosing intervals and hybrid immunity have all been shown to shape the B cell reservoir, recall response, and repertoire diversity.

The lessons learned from the COVID-19 pandemic underscore the importance of early identification of CoPs, adaptable and high throughput monitoring platforms and understanding diverse immune responses to improve vaccine development against future pathogens.
